# Comparative analysis of miRNAs of two rapeseed genotypes in response to acetohydroxyacid synthase-inhibiting herbicides by high-throughput sequencing

**DOI:** 10.1371/journal.pone.0184917

**Published:** 2017-09-26

**Authors:** Maolong Hu, Huiming Pu, Jianqin Gao, Weihua Long, Feng Chen, Wei Zhang, Xiaoyin Zhou, Qi Peng, Song Chen, Jiefu Zhang

**Affiliations:** Institute of Industrial Crops, Jiangsu Academy of Agricultural Sciences / Nanjing Sub-center, National Center of Oil Crops Improvement / Key Laboratory of Cotton and Rapeseed (Nanjing), Ministry of Agriculture, Nanjing, China; National Botanical Research Institute CSIR, INDIA

## Abstract

Acetohydroxyacid synthase (AHAS), also called acetolactate synthase, is a key enzyme involved in the first step of the biosynthesis of the branched-chain amino acids valine, isoleucine and leucine. Acetohydroxyacid synthase-inhibiting herbicides (AHAS herbicides) are five chemical families of herbicides that inhibit AHAS enzymes, including imidazolinones (IMI), sulfonylureas (SU), pyrimidinylthiobenzoates, triazolinones and triazolopyrimidines. Five AHAS genes have been identified in rapeseed, but little information is available regarding the role of miRNAs in response to AHAS herbicides. In this study, an AHAS herbicides tolerant genotype and a sensitive genotype were used for miRNA comparative analysis. A total of 20 small RNA libraries were obtained of these two genotypes at three time points (0h, 24 h and 48 h) after spraying SU and IMI herbicides with two replicates. We identified 940 conserved miRNAs and 1515 novel candidate miRNAs in *Brassica napus* using high-throughput sequencing methods combined with computing analysis. A total of 3284 genes were predicted to be targets of these miRNAs, and their functions were shown using GO, KOG and KEGG annotations. The differentiation expression results of miRNAs showed almost twice as many differentiated miRNAs were found in tolerant genotype M342 (309 miRNAs) after SU herbicide application than in sensitive genotype N131 (164 miRNAs). In additiond 177 and 296 miRNAs defined as differentiated in sensitive genotype and tolerant genotype in response to SU herbicides. The miR398 family was observed to be associated with AHAS herbicide tolerance because their expression increased in the tolerant genotype but decreased in the sensitive genotype. Moreover, 50 novel miRNAs from 39 precursors were predicted. There were 8 conserved miRNAs, 4 novel miRNAs and 3 target genes were validated by quantitative real-time PCR experiment. This study not only provides novel insights into the miRNA content of AHAS herbicides tolerant rapeseed in response to AHAS herbicides, but also demonstrates that miRNAs may be involved in AHAS herbicides tolerance.

## Introduction

Acetohydroxyacid synthase (AHAS, EC4.1.3.18), also called acetolactate synthase (ALS, EC2.2.16), is a key enzyme which plays a vital role in the first step of the biosynthesis of the branched-chain amino acids valine, isoleucine and leucine. AHAS catalyzes two parallel reactions: the condensation of two pyruvates to generate acetolactate, and 2-ketoglutarate to generate acetohydroxybutyrate. Substantial research on *Arabidopsis*, barley and sunflower showed that some base substitution in resistant mutants led to changes in amino acids which resulted in interference with herbicides acting on AHAS enzymes [1,2]. There are five chemical families of herbicides that inhibit the AHAS enzyme, including the imidazolinones (IMI), the sulfonylureas (SU), the pyrimidinylthiobenzoates (POB), the triazolinones (TZ, also known as sulfonylaminocarbonyltrazolinones) and the triazolopyrimidines (SCT), thus these herbicides could be called Acetohydroxyacid synthase-inhibiting herbicides (AHAS herbicieds). The SU and IMI herbicides have been in use over two decades [3]. SU herbicides (chlorosulfuron, metsulfuron-methyl and triasulfuron) are cheap, effective and safe herbicides that act against a wide spectrum of weeds at low rates of application, but their breakdown is very slow [4,5]. They can inhibit the activity of AHAS through binding to a quinone-binding site. IMI herbicides are registered for use on non-pulse crops including rapeseed, sunflower, barley, spring wheat, oats, and pulse crops: lentil, dry bean, field pea and soybean [6].

Since their first identification in *Caenorhabditis elegans* in 1993, miRNAs have been found to be key post-transcriptional regulators of eukaryotic gene expression [7]. In plants, miRNAs are fundamental, sequence-specific regulatory elements in the genome [8]. miRNAs are usually encoded by 20 to 24 nucleotides (nt), like some other small RNAs, but differ in biogenesis, precursor structures and modes of action. After fully or partly pairing with complementary sequences, especially 3′ untranslated regions of target mRNAs to guide RISCs, the miRNA may repress the target transcript through translational inhibition, accelerated exonucleolytic mRNA decay or slicing within miRNA-mRNA base pairing [8,9]. Many comprehensive databases and websites have been built to store and analyze the quickly growing miRNA information. Among them, the miRBase (http://www.mirbase.org/) is the primary microRNA sequence repository. It contains 28645 entries, representing hairpin precursor miRNAs, expressing 35828 mature miRNA products in 223 species. Its updated release assigns confidence levels for each miRBase entry in accordance with deep sequencing data, providing users with more accurate expression patterns [10].

The miRNAs of rapeseed have been identified and characterized in several studies. Using the computational method, a total of 21 potential miRNAs were identified based on previously known miRNAs against expressed sequence tags and genomic survey sequences at that time [11]. By constructing a library of small RNA sequences, 11 conserved miRNA families were identified and confirmed by secondary structure prediction. The miR169 family was highly expressed in young leaves and stems but not in roots and mature leaves, supporting the hypothesis that miR169 plays an important role in development [12]. Systematic analysis identified 50 conserved miRNAs and nine novel miRNAs, and the expression pattern of some members showed significant differences in several embryonic development stages or in different seed oil content cultivars [13]. To date, the miRBase version 21 contains 90 highly confident mature miRNA products identified from rapeseed. These studies and data provide useful resources for novel miRNA identification, target gene searches, comparative analysis and functional characterization of miRNAs in rapeseed. In addition, there were also a large number of studies focused on the genome-wide analysis of miRNAs and tasi-RNAs in variety developmental phases and in response to stresses. A total of 97 conserved and 526 novel miRNAs were identified from sorghum, of which unique miRNAs could be regulated by drought stress [14]. A recent study on maize reported 174 known and conserved differentially expressed miRNAs and 155 new families [15].

Based on the *Arabidopsis* AHAS genes, the AHAS amino acid substitutions can cause herbicide tolerance at the Ala122, Pro197, Ala205, Asp376, Trp574, and Ser653 positions [16,17]. Mutations at different positions of AHAS genes can result in differentiated resistance to different AHAS herbicides. In recent years, research on those herbicides mainly focused on germplasm resources and enhancement, gene cloning and function verification. To the best of our knowledge, this is the first report of small RNAs identification and characterization of two rapeseed genotypes in response to AHAS herbicides using high-throughput sequencing technology. We applied this technology to achieve comparative profiles of miRNAs with the aim of identifying the miRNAs expressed differentially in AHAS sensitive genotype and AHAS tolerant genotype after spraying SU and IMI herbicides. These differential expressed miRNAs might play a vital role in AHAS herbicide resistance pathways.

## Materials and methods

### Plant materials and herbicide application

As part of this study, rapeseed genotypes were screened based on their phenotypic response to varying level of treatments by applying AHAS herbicides as outlined below. Based on their morphological responses, M342 was selected as the AHAS-tolerant material due to its cross-tolerance to both IMI and SU herbicides. The genotype N131 was selected as a control that was sensitive to SU and IMI herbicides and shared a close background with the genotype M342. Seedlings of these two genotypes were grown in 30-cm diameter plastic pots (six plants per pot) which contained a 1:1:1 mixture of peat moss, perlite and vermiculite. The seedlings were kept in a glasshouse, at 22–25°C with a 16h light/8h dark photoperiod and relative humidity of 80%. A group of 1-month-old rapeseed seedlings were sprayed using 5.0% Douleshi IMI herbicide (Shangdong Syngenta Ltd.) and 10% tribenuron-methyl wettable-powder SU herbicide according to the practical farming rules, while the control seedlings of these two genotypes were treated with equal amounts of water. Fresh leaves of these seedlings were collected at three time points: 0h, 24h and 48h after herbicides spraying. These leaves were frozen immediately in liquid nitrogen and then stored at -70°C for further total RNA extraction.

### small RNAs library construction and sequencing

Total RNAs were extracted using Trizol reagent following the manufacturer’s instructions. A NanoDrop ND-20000 spectrophotometer (Thermo Fisher, Waltham, USA) was used to check total RNA quantity and purity of all 20 samples at 260/280 nm (ratio>2.0). The RNA balanced mix samples were fractionated by 15% denaturing polyacrylamide gel electrophoresis, and then the small RNA fragments between 18 and 30 nt were isolated from the gel and ligated to a 5’ adaptor and a 3’ adaptor by T4 RNA ligase. The small RNA libraries were sequenced with Illumina HiSeq^™^ 2500 by Genepioneer Biotechnologies Co, Ltd., Nanjing, yielding millions of reads of around 51nt in length, including the adapters. The adapters were removed automatically by the sequencing machine with default parameters. All small RNA sequencing data files (fastq format) are available from the NCBI GenBank Database under the accession number PRJNA338834.

### Classification of small RNAs

Strict quality trimming and adaptor removal of the Illumina reads were processed by a Perl script. First, low quality reads containing even one base with a quality score below 20 were discarded. Adapters and poly-A fragments were trimmed and then only 17–46 nt sequences were retained. The resulting clean reads were processed into non-redundant cluster sequences, and then aligned to the latest *B*. *napus* genome (http://www.genoscope.cns.fr/brassicanapus/, v5) using bowtie and no gap and at most 1 mismatch were allowed in alignment (parameters: bowtie -v 1 -a -m 1 --best --strata --norc–sam) [18], and then sorted and viewed by SAMTools (parameters: samtools view -b -h -F 4 –S) [19]. Furthermore, the alignment regions on the rapeseed genome were analyzed using ANNOVAR software to characterize the distribution of reads on exons, introns, splicing sites and intergenic regions of rapeseed genes.

The rRNA, snRNA and snoRNA databases were obtained from Rfam (http://rfam.xfam.org/, version 11.0) [20,21]. The tRNA databases were gathered from UCSC (http://gtrnadb.ucsc.edu/) [22] and Rfam. The reads in clean datasets were aligned against these databases using bowtie and assigned the potential classification to these reads. Only the best-matched alignment was considered as the potential classification. The ta-siRNAs of 16 plants were downloaded from PNRD (Plant Non-coding RNA Database, http://structuralbiology.cau.edu.cn/PNRD/) [23]. The reads were then aligned with those ta-siRNAs to predict known conserved ta-siRNAs, according to a previous report [15], while no mismatch was allowed in this study.

### Identification of conserved and novel miRNAs

The remaining reads after non-coding RNA classification (except miRNAs) were aligned with all mature reads of plant miRNAs in miRBase (http://www.mirbase.org/, version 21) [10]. The reads with a length of 20 to 24nt were considered. Moreover, the Mireap software (http://sourceforge.net/projects/mireap/, v2.0) was used for prediction of novel miRNAs. The prediction results were valued and only those that met the following criteria were regarded as potential miRNAs: (1) the iso-miR were excluded; (2) the secondary structure of miRNA precursor was stable, and the minimum free energy (MFE) was lower than -20 kcal/mol; (3) the potential miRNA was located in intergenic regions; (4) the miRNA was assigned with high-confident miRNA if its precursor shared similarity with known miRNAs [24,25]. In addition, the secondary structure of novel miRNAs in rapeseed were predicted using RNAfold (http://rna.tbi.univie.ac.at/, parameters: RNAfold -p -d2 --noLP).

### Differential expression analysis of miRNAs

The frequency of miRNAs was normalized to TPM (number of Transcripts Per Million clean tags). To allow for the fact that some regions on the same arm may also be expressed simultaneously with mature miRNA, two types of alignments were counted into the expression of miRNAs: (1) the reads completely contained reference miRNA in miRBase and were 4nt longer than mature miRNA at most; (2) the reads located on the same arm as mature miRNA and having at most three mis-matches with reference miRNA were allowed.

The two sequenced replicates were considered as one group, and the expression of miRNAs was compared among groups. The differentially expressed miRNAs were screened in accordance with previous reports. The miRNAs with a log_2_ value (Fold Change) larger than 1 (in another words, down-regulated or up-regulated outweighed twice that of the others) and a p-value smaller than 0.05 were considered to be differentially expressed miRNAs [26]. The miRNAs were considered to be very significantly different and significantly different if the corresponding p-values fell within 0–0.01 and 0.01–0.05 respectively.

### Validation of miRNA expression

We performed qPCR to explore the validation and stress expression patterns of identified miRNAs. The expression of selected miRNAs was assayed in rapeseed by Platinum SYBR Green based qPCR (Invitrogen, 11733–038) with the High Specificity miRNA QuantiMir RT Kit (RA610A-1, System Biosciences) on Step One^™^ Real-Time PCR System (Applied Biosystems). A total of 10 conserved miRNAs, 4 novel miRNAs and 3 corresponding target genes were validated by qPCR technology. These miRNAs and the internal control genes (5.8S rRNA) are available in Table A in S1 File. The qPCR reactions were performed as follow: 94°C for 30 s, and then 40 cycles at 94°C for 10 s, 58°C for 30 s. All the gene expression data were obtained from three individual biological/technical replicates and processed according to previous analyses [27,28].

## Results and discussion

### Substitutions of AHAS genes in rapeseed and resistance change

Herbicides are extremely effective tools for weed control. The present study was undertaken with the objective of understanding the nature of inheritance and the molecular basis of AHAS herbicides tolerance in the cultivar M342. There are several commercially available rapeseed, including two mutants M9 and PM1 at the BnAHAS1 (*Brassica napus* AHAS1) locus. The RT-PCR confirmed that with the substitution mutation (SER653Asp) in the M9 genotype, the transcription of BnAHAS1 was neither up-regulated nor down-regulated compared with the wild type. Using EMS-induced mutagenesis and directional breeding technology, a novel SU-insensitive mutant named M342 was obtained and was sequenced in this study (Chinese Patent, 201310054645.9). After spraying SU herbicide, M342 grew normally without any injury symptoms. Further gene cloning showed that there was one base substitution (G to T in position 1688), resulting in a single amino acid change (557 Tryptophan to Leucine substitution) within a region of BnAHAS3. This is entirely consistent with the PM2 mutation of rapeseed described in a previous report [29]. The corresponding substitution in *Arabidopsis* is Trp574. This study was conducted to study the growth conditions and RNA network regulation at the gene level of this novel genotype. After spraying the herbicides, the interior leaf of the sensitive N131 rapeseed plants turned yellow seven days after spraying the herbicides, and then died in fourteen days. At the same time, the tolerant M342 rapeseed plants grew normally, showing its high herbicide resistance and that it could be used as a valuable commercial breeding material.

### Overview of small RNA sequencing

To examine small RNAs associated with the tolerance of AHAS herbicides of two genotypes in rapeseed, deep sequencing of small RNAs was used to construct 20 small RNA libraries from leaves of tolerant and sensitive genotypes resistant to SU and IMI herbicides. The total sequence reads were taken after adaptor removal by the sequencing machine.

**Fig 1:**
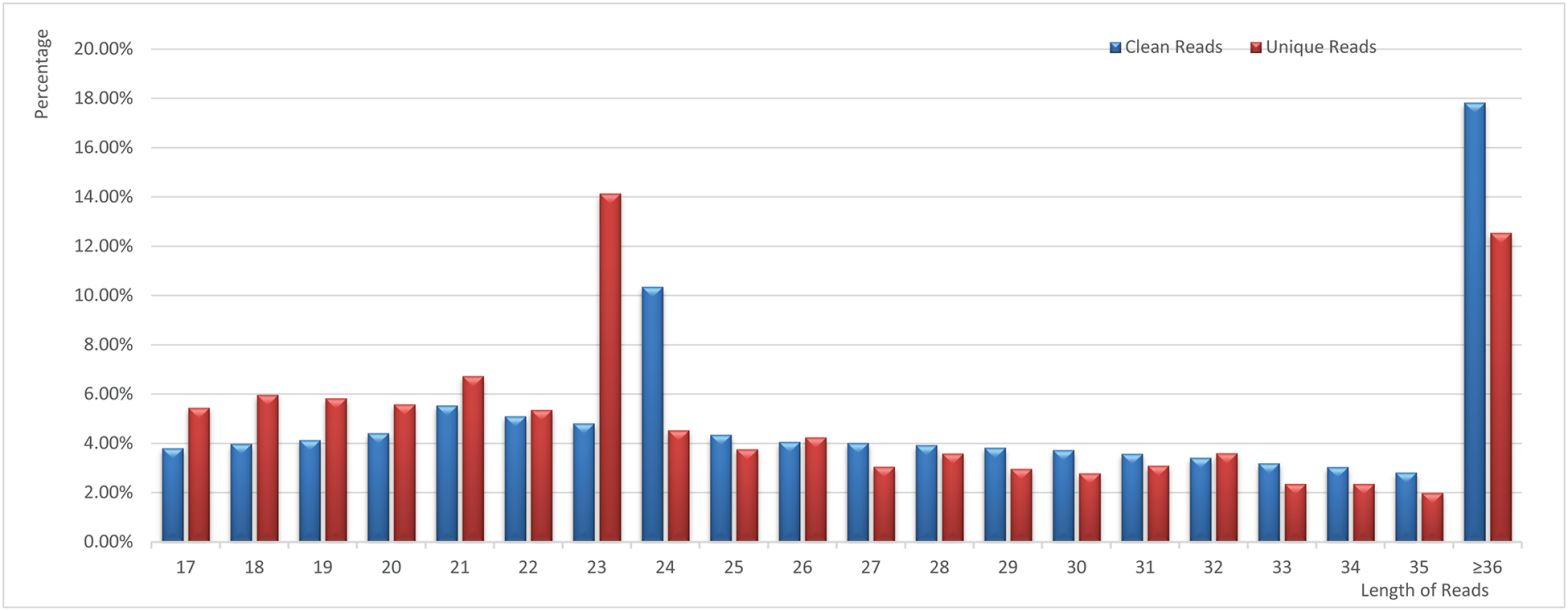
Length distribution of redundant and non-redundant small RNA sequences.

To investigate small RNAs involved in AHAS enzyme herbicide, high-throughput sequencing was used to identify and characterize small RNAs in 20 samples, yielding a total of 231 million reads. The number of reads in each sample ranged from 10.05 million to 16.02 million reads, which were sufficient to identify and characterize the expression of small RNAs. The length of small RNAs varied widely, ranging from 17 to 46 nt. In these 20 small libraries, the major classes of total small RNAs were 24nt in length, followed by 21nt. In addition, the 24nt small RNAs were the dominant type in non-redundant small RNA sequences, which was consistent with previous results in non-heading Chinese cabbage [27] (Table B in S1 File, Fig 1).

### Classification and distribution of all small RNAs

To investigate which kind of small RNAs are involved in the tolerance to acetolactate synthase herbicides in rapeseed, all the sequenced data were classified into different types according to well-characterized databases such as Rfam and UCSC (Table C in S1 File). In *Arabidopsis thaliana*, comparisons of the results of Illumina sequencing reads from either total RNA or gel purified RNA showed there was an increase in reads mapping to tRNAs from 0.0007% to 13.58%, respectively, with the ratio of rRNA found to decreased dramatically [30]. A total of around 30% and 2.8% reads were annotated as rRNA and tRNA respectively, which is similar with the ratio of 19.6% and 0.7% in another research [13]. rRNA represented the most abundant type of small RNAs, but the percentage of rRNA in all small RNAs in this study might be underestimated because rRNAs were eliminated as much as possible during the RNA sample preparation procedure. In addition, snoRNA, miRNA and snRNA represented about 6.5%, 5.8% and 0.8% in all clean reads, respectively (Table 1). We also found three ta-siRNAs in rapeseed, which were resulted from TAS3 genes (Table 2).

**Table 1:** The region assigned for reads and types of small RNAs.

classification	N1_2	N4_5	N7_8	N10_11	N13_14	M1_2	M4_5	M7_8	M10_11	M13_14
upstream/downstream	3913511	4009427	3893649	3852407	3592189	3321309	3997889	4621568	3163676	4131469
exonic	5814929	6157212	6956709	5491859	5065973	5514465	5956750	7588615	4217674	4581691
intergenic	11099224	12499293	9604345	13143709	12648064	11476432	10965642	13327593	11630309	13688212
intronic	654858	696752	559603	733323	623163	709262	739626	743417	589579	723438
splicing	19109	20170	16383	19155	16279	20239	46378	23205	15236	16509
UTR	71627	65605	59173	65620	58923	62172	53389	70436	44888	53083
all	22246070	24218464	21694757	24161957	22654114	21982583	22625767	27150131	20335834	23785019
miRNA	1448794	1380801	1034619	1408550	1334057	1225323	1336438	1416535	1314056	1547498
tRNA	904401	624897	600145	806166	723640	100611	845289	523526	461858	886839
rRNA	6473408	7381713	5560193	7825892	7053448	7421646	5869438	7902664	6164975	7588608
snRNA	177209	190981	139020	213632	195535	132864	154372	219400	209833	237380
snoRNA	1219809	1747467	1149380	1713354	1757764	767004	1055036	1377053	1934783	2353905
Others	12022449	12892605	13211400	12194363	11589670	12335135	13365194	15710953	10250329	11170789

**Table 2:** The ta-siRNA found in rapeseed and their expression.

Name	Sequence	N1_2	N4_5	N7_8	N10_11	N13_14	M1_2	M4_5	M7_8	M10_11	M13_14
atTAS3a-5-D7;oeTAS3a-5-D7;oeTAS3b-5-D7;slTAS3-5-D7	TTCTTGACCTTGTAAGACCCC	0.18	0.12	0.00	0.17	0.44	0.05	0.22	0.03	0.39	1.01
vvTAS3-tasiRP7a	TTCTTGACCTTGTAAGACCCT	0.04	0.08	0.00	0.04	0.00	0.00	0.18	0.04	0.15	0.53
bnTAS3a-5-D7;bnTAS3b-5-D7	TTTCTTGACCTTGTAAGACCC	0.09	0.00	0.14	0.04	0.35	0.05	0.13	0.09	0.19	0.67

A large number of small RNAs were found to be associated with genomic repeat regions. The clean reads were compared with repeat regions in the RepeatMasker Database to characterize the repeat types and amount in the rapeseed genome. In total, 18.75 million reads were identified to be simple repeats and low complexity repeats, followed by the SINE, LINE and LTR regions. The SINEs are non-functional repeats of RNA genes which have been reintegrated into the genome with the assistance of a reverse transcriptase (Table D in S1 File). Among the SINEs found in this study, the SINE/Deu was the most abundant type, followed by the SINE/MIR, SINE/tRNA subfamilies.

The specific distribution region of small RNAs on genes were potentially associated with the function of small RNAs. In this study, over half of small RNAs were located in the inter-genic regions, followed by exonic regions (24.84%) and UTR regions (16.68%). Less than 1% of small RNAs were located in exon-intron splicing regions and UTR regions (Table C in S1 File).

### Known miRNAs in rapeseed and annotation

Conserved miRNA identification always relies on information in miRBase, which contains 184 rapeseed miRNAs to date. In contrast, there are 760 miRNAs from *Arabidopsis* stored in miRBase, although the real number of miRNAs in *Arabidopsis* is less than in rapeseed due to a smaller genome. Thus, we collected 8164 miRNAs from verified plants as a dataset, and then aligned sequenced reads to these miRNAs to identify the expression of miRNAs. As known rapeseed miRNAs were identified according to information of plant miRNAs in miRBase, they were named after their counterparts from other reference plants.

Use of this computational method showed 940 miRNAs belonging to 430 families were identified in all samples (Table E in S1 File). To obtain a global view of the abundance of these conserved miRNAs, the total number of each miRNA from 20 samples were calculated, and are shown in Table F in S1 File. The miR156 family contained 46 conserved miRNAs in rapeseed, which was the biggest family-contained maximum. The average expression of members in the miR398 family reached 474.46 TPM, which indicates its important role in rapeseed. This was followed by the miR166 and miR159 families, with an average expression of 396.67 and 269.42 respectively. Interestingly, only one miRNA belonged to the miR5083 family, but had an expression reaching 10.68 TPM.

Furthermore, 3284 genes in rapeseed had been predicted as targets of 510 miRNAs (Table G in S1 File). Of these genes, 35 were predicted to be targets of over 20 miRNAs in rapeseed. According to our results, a large number of miRNAs might regulate multiple genes, and one gene also could be regulated by multiple miRNAs. For instance, ath-miR414 could possibly regulate 102 genes in rapeseed. However, no AHAS gene in rapeseed was predicted to be a target of these miRNAs, indicating that miRNAs might not play a direct role in handling AHAS-related herbicide stress.

**Fig 2:**
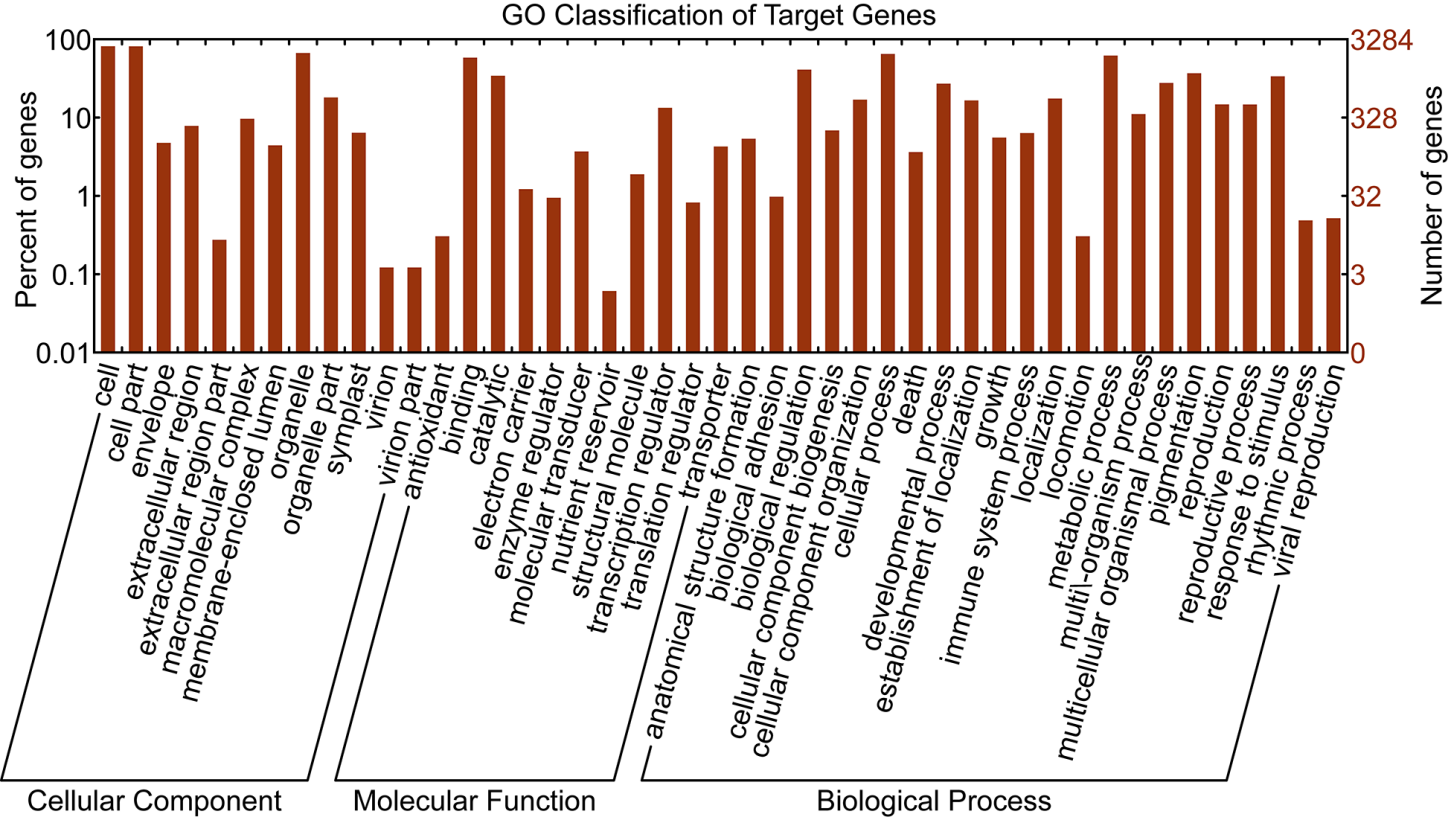
GO classification of genes that target known rapeseed miRNAs.

To obtain deeper insight into what pathways these miRNAs may be involved in, we annotated these target genes with the GO and KOG databases. There were 3020 genes which were annotated based on the best BLASTX hit with GO terms. The GO classification showed that Cell, Cell Part in the Cellular Component supergroup and Cellular Process in the Biological Process supergroup were the top three groups, indicating that these target genes were highly associated with cell structure and pathway functions (Fig 2).

**Fig 3:**
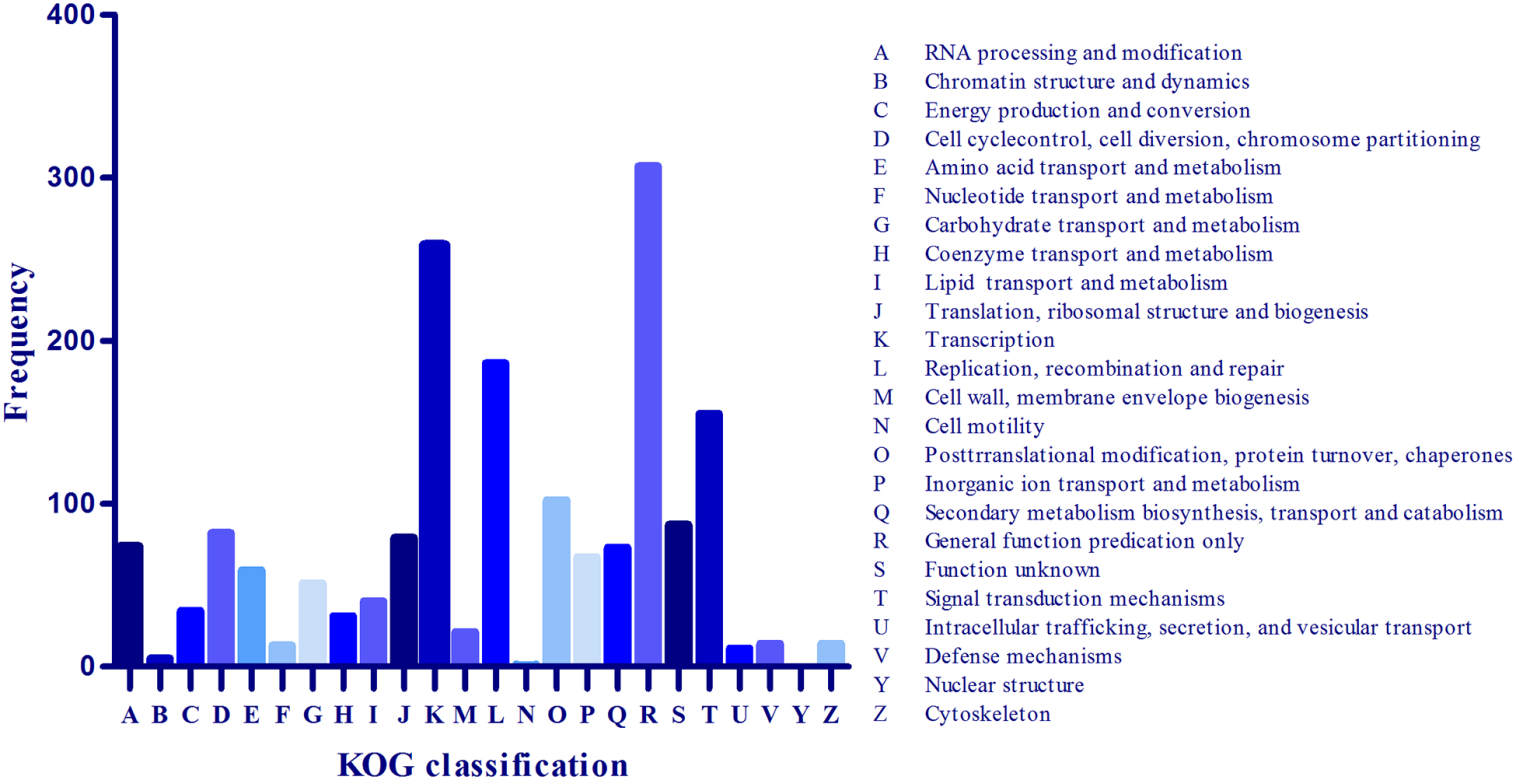
KOG classification of genes that target known rapeseed miRNAs.

The euKaryotic Ortholog Group (KOG) terms were assigned to each target gene based on the best BLASTX hit from the KOG database. The KOG database contains detailed functional and evolutionary patterns in 5,873 clusters of predicted orthologs among seven eukaryotic genomes [31]. Out of 3284 genes, 1678 were assigned into 23 KOG groups. The R subgroup (General Function Prediction) was the largest, containing 307 genes, followed by the K subgroup (Transcription, 258 genes) and L subgroup (Replication, Recombination and Repair, 1799 genes) (Fig 3).

### qPCR validation

A qRT-PCR analysis was performed to detect the relative expression levels of six miRNAs in response to SU and IMI herbicides. In contrast to high-throughput sequencing, we detect the relative expression levels of samples only at 48h after herbicide implication. The correlation between the results was evaluated in R language using the RNA-seq fold-change values and relative expression levels quantified by qPCR. In order to normalize data, the average of relative expression levels of samples by qPCR were used to compare with 5.8S rRNA to calculate fold-change values (Table A in S1 File). The expression of osa-miR5083 increased to 14.49-fold and 7.87-fold at 48 h after spraying SU and IMI herbicides in N131 genotype. The expression of osa-miR5083 also showed similar expression patterns in M342 genotype, and increased to 6.92-fold at 48h after spraying IMI herbicides compared with 0 h. The expression patterns of bna-miR6029, ahy-miR159 and cpa-miR8155 were also increased in both N131 and M342 genotype after herbicide treatment. However, the expression patterns of aau-miR396 were different, as it insignificant up-regulated after SU herbicide treatment while it up-regulated over 11-fold after IMI herbicide treatment in N131 genotype. The expression of vvi-miR396b was up-regulated in these two herbicides after herbicide treatment. The aly-miR396a-3p were relatively steady in N131 after treatment, while its expression almost increased over 7.5-fold at 48 h after SU and IMI herbicide spraying. The expression of aqc-miR166a was up-regulated after IMI herbicide treatment in both genotypes, whereas only up-regulated after SU herbicide treatment in N131 genotype.

For the four novel miRNAs, the expression of bna-miR-n029-5p was up-regulated almost 13-fold after IMI and SU herbicide treatment in N131 genotype. However, the expression of bna-miR-n029-5p down-regulated 0.86-fold in M342 genotype, whereas it insignificant expressed at 48h after SU herbicide. The expression of bna-miR-n001-3p was up-regulated after IMI and SU herbicide treatment in N131 genotype, but insignificantly expressed after SU herbicide treatment. The expression of bna-miR-n002-5p was up-regulated after SU herbicide while down-regulated after IMI herbicide treatment in N131 genotype, while the regulation tendency was reversed in M342 genotype. The expression of bna-miR-n003-5p was insignificantly expressed after SU herbicide while down-regulated after IMI herbicide treatment in N131 genotype, while they were both down-regulated in M342 genotype.

Furthermore, in order to give insight into the function of miRNAs in post-transcriptional level, three target genes were validated by qPCR. The GSBRNA2T00051008001 was encoded thioredoxin, which was targeted by aly-miR396b-5p by prediction. The qPCR results showed that its expression down-regulated over 10-fold after SU or IMI herbicides treatment in N131 genotype, and also down-regulated in M342 genotype. Interestingly, the expression of aly-miR396b-5p had an up-regulation trend after herbicides treatment by RNA-seq technology, which supported aly-miR396b-5p could *cis*-regulate GSBRNA2T00051008001. One of the target genes for bna-miR-n017, GSBRNA2T0013464600, encoding Homeodomain-like (DEK) protein. The expression of GSBRNA2T0013464600 down-regulated slightly after herbicide treatment in N131 genotype, while it down-regulated significantly at 48 h after SU herbicide treatment. A calmodulin-binding protein termed GSBRNA2T00114351001 was also validated, which could be a target of the novel miRNA bna-miR-n029. The expression of this gene significantly down-regulated at 48 h after IMI herbicide treatment, while insignificantly up-regulated or down-regulated in other treatments.

### Novel miRNAs in rapeseed

Novel miRNA candidates were selected based on the secondary structure of precursor sequences, the miRNA/miRNA* duplex and the minimal folding free energy (MFE) value. A large dataset containing 3073 possible miRNA precursors was predicted by Mireap software. After the MFEI and secondary structure check of precursors, 39 of them were retained as potential candidates as miRNA precursors for further analysis, representing 50 mature miRNAs. Over half (51.02%) of the novel miRNAs were located in the 5’ arm of the hairpin structure. The MFE of novel miRNAs varied from -18.4 (kcal/mol) to -61.0 (kcal/mol) (Table H and Table I in S1 File). The secondary structure of novel miRNAs were shown in S2 File. The MFEI index of these novel miRNAs were ranged from 0.7 to 1.65. To identify the novel miRNAs associated with AHAS herbicide tolerance capacity, the expression of novel miRNA candidates was calculated after novel miRNA identification.

### miRNA families response to SU herbicide

**Fig 4:**
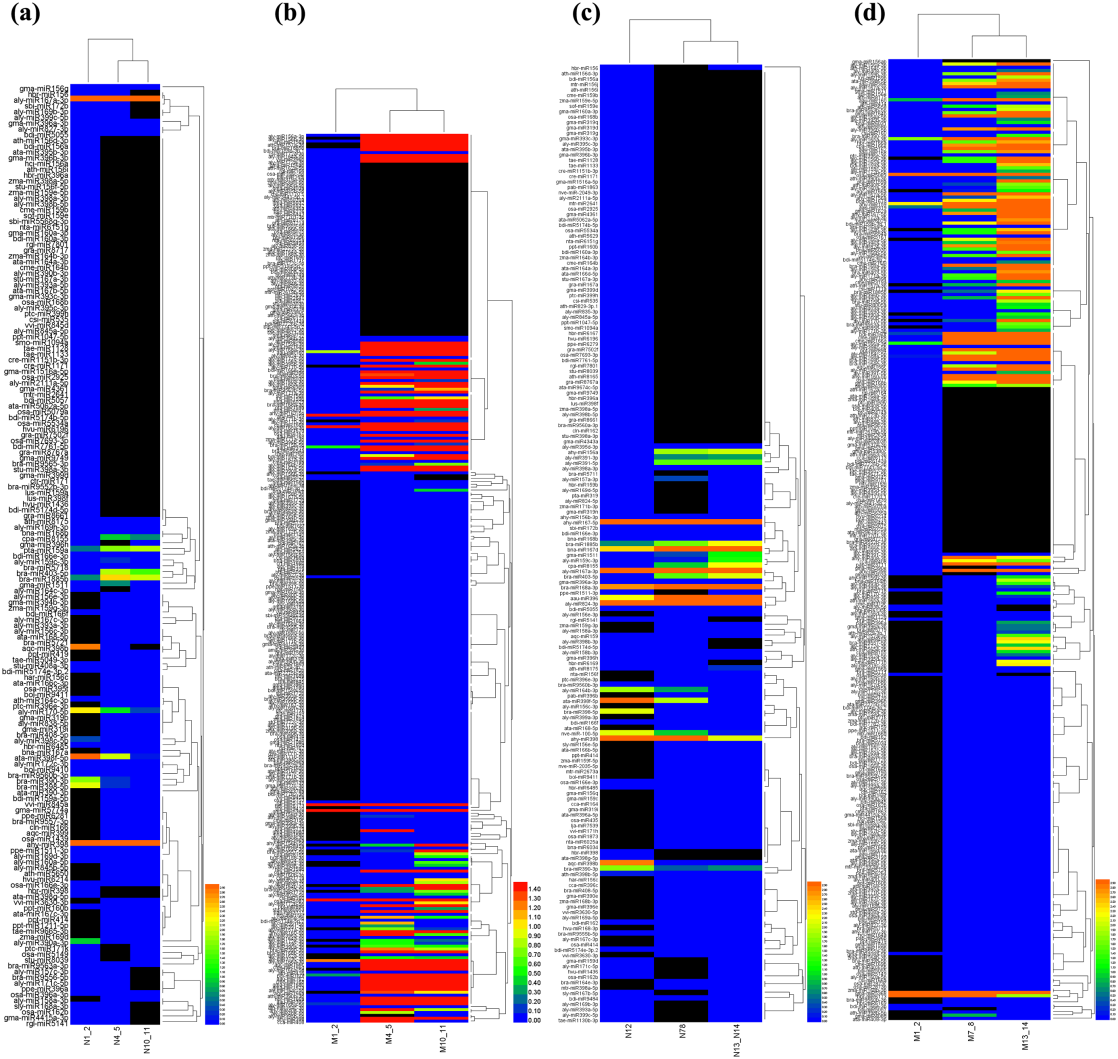
The heat map of differentiated miRNAs after spraying herbicides in both sensitive and tolerant genotypes. (a) SU herbicide treatment in genotype N131; (b) SU herbicide treatment in genotype M342; (c) IMI herbicide treatment in genotype N131; (d) IMI herbicide treatment in genotype M342.

In the presence of SU herbicide, the expression of most differential miRNAs increased in M342 genotype while it decreased in CK (control) at the same time (Fig 4a and 4b). For instance, the initial expression value of ahy-miR398 was around 4535.53 TPM in CK before applying herbicide. After treatment with herbicide, the expression sharply declined to 12.49 TPM in 24h after spraying and then maintained steadily to 48h. The other members of the miR398 family had low expression levels before spraying and their expression also decreased sharply after spraying. The expression pattern was different in M342 samples. The initial expression before spraying was around 0.88 TPM, which was 0.02% compared with CK. Interestingly, the expression went up to 60.67 TPM at 24h and went down to 3.32 TPM at 48h. Other members in the miR398 family showed similar expression patterns, as their expression increased at 24h and 48h after spraying compared with in the absence of SU herbicide. The miR398 family was proposed to be directly linked to the plant stress regulatory network and regulates plant responses to oxidative stress, water deficit, salt stress, abscisic acid stress, ultraviolet stress, copper and phosphate deficiency, high sucrose and bacterial infection [32]. The ahy-miR398 in peanut was first identified in 2010 and hypothesized to regulate serine hydroxymethyltransferase [33,34]. In addition to those functions, this study indicates that miR398 members are also response to SU herbicide and might involve in herbicide tolerance.

### miRNA families response to IMI herbicide

In accordance with the miRNA expression pattern after spraying SU herbicides, the expression of almost 80% of differential miRNAs increased in M342 while most decreased in CK at the same time (Fig 4c and 4d). The miR395 members expressed at a low level remained steady in CK, but increased sharply in the M342 genotype after IMI herbicide spraying. For instance, the expression value of aly-miR396b-5p was initially 0.27 TPM. After spraying IMI herbicide, its expression increased 24.63 times to 6.68 TPM, then kept increasing to 27.53 TPM at 48h after spraying. Other members of the miR396 family, including aau-miR396, vvi-miR396b, aly-miR396a-3p and aly-miR396b-3p, also increased over 10 times after spraying. Some relatively conserved miRNAs tend to correspondingly increase in both M342 and N131 genotypes after spraying, such as cpa-miR8155. The miR8155 family was first identified in papaya and was also confirmed in tomato [35,36]. However, the function of its members has not been identified. This study shows that miR8155 might play a role in herbicide resistance or some other stress. The qPCR results showed that rapeseed miR8155 kept increasing after SU and IMI treatment at 24h and 48h in sensitive N131 genotype. However, in tolerant M342 genotype, the miR8155 increased 4-fold at 24h after SU treatment and then halved at 24h, while it slightly down-regulated at 24h after IMI treatment and then increased 3-fold compared with control.

### Differentiated miRNAs of tolerant genotype after SU herbicide application

In order to characterize the miRNAs that participate in tolerance pathways to SU herbicide, differentiated miRNAs were selected from AHAS herbicides sensitive and tolerant genotypes according to their significance and likelihood (Table J and Table K in S1 File). To control the bias that may result from the high-throughput sequencing method, two biological replicates were adopted in this study. Differentiated miRNAs were identified when significance differences exist in different controls while no significant difference exists in two replicates. In order to obtain more conserved miRNAs that are related to AHAS tolerance and to reduce false positivity, only mature miRNA sequence that were found in at least one plant species were used to characterize the differentiated miRNAs.

164 miRNAs were defined to be differentiated miRNAs in sensitive genotype N131 after SU herbicide application. In contrast, almost twice as many differentiated miRNAs (309) were found in tolerant genotype M342 after SU herbicide application. Larger numbers of differentiated miRNAs may indicate possible complex mechanisms responding to SU herbicide. Among these miRNAs, the expression of 64 miRNAs were simultaneously differentially expressed in these two genotypes, showing that they potentially have no function in relation to SU herbicide tolerance. A substantial number of miRNAs was detected after SU herbicide application while nothing were detected before treatment. For example, the high-throughput sequencing data showed the expression of aly-miR166a-5p and aqc-miR166a up-regulated and reached 7.31 TPM at 24h, and kept steady at 48h after SU application. The aqc-miR166a may regulate 13 rapeseed genes, which needs further validation.

### Differentiated miRNAs of tolerant genotype after IMI herbicide application

The differentiated miRNAs that are involved in IMI herbicide stress response were also detected (Table L and Table M in S1 File). There were 177 and 296 miRNAs defined as differentiated in sensitive genotype N131 and tolerant genotype M342 after IMI herbicide application, respectively. The differentially expressed miR156/157 members in genotype N131 had low expression, whereas their expression went up dramatically in genotype M342 after IMI herbicide application. The miR398 family was also expressed differentially in two genotypes. The expression of miR398 members was high in N131 before IMI herbicide application, and then went down to 1–10% of the initial value. However, the expression of miR398 members increased several times in tolerant genotype M342.

## Discussion

There are one or several copies of AHAS genes in some plants. CSR1 (AT3G48560) is the sole gene that codes for AHAS in *Arabidopsis thaliana*. An *Arabidopsis* mutation csr1-2 (Ser-653-Asn) confers selective resistance to the imidazolinone herbicides, and it has been shown that AHAS is the sole target of imazapyr by transcriptome analysis [37]. Another lethal null mutant, csr1-7, was generated by a T-DNA insertion into the CSR1 gene. The comparison of these substitution mutants that are generated through different methods show that transgenic lines yield significantly higher levels of resistance and greater biomass accumulation in the presence of imazapyr than the mutagenic line csr1-2. Microarray analysis has shown few differences in their transcriptomes, except for sevenfold to tenfold transcription increase of csr1-2, which may contribute to the different levels of herbicide resistance [38]. A similar situation was also confirmed in chickpea. qPCR analysis indicated that there is no significant change in transcription levels of chickpea AHAS1 between sensitive and tolerant genotypes after IMI herbicide application [6].

Early in 1991, five members of the AHAS gene family were identified in the rapeseed cultivar Topas [39], while four of them were cloned and sequenced in 1992 [40]. DNA sequence analysis indicated that BnAHAS1 and BnAHAS3 shared extensive homology, while BnAHAS2 has diverged significantly from BnAHAS1 and BnAHAS3 in the coding region of the mature polypeptide, transit peptide and upstream non-coding DNA. In contrast, BnAHAS4 and BnAHAS5 have interrupted coding regions and may be defective [39]. The whole genome sequencing of rapeseed confirmed that there are five AHAS genes in the genome, including GSBRNA2T00078037001, GSBRNA2T00125907001, GSBRNA2T00132697001, GSBRNA2T00138520001 and GSBRNA2T00139270001. In this study, no miRNA has been predicted to directly regulate these five genes in rapeseed. However, hundreds of miRNAs were found to be associated with gene expression regulation after herbicide spraying, showing differential expression patterns.

A transgenic *Arabidopsis thaliana* genotype was generated conditionally expressing a hairpin dsRNA construct of a mutated acetolactate synthase gene coding sequence, which confers chlorsulfuron tolerance in the presence of dexamethasone (DEX). The suppression of the endogenous AHAS mRNA expression and 21-nt siRNA expression were detected after DEX application. The results showed that AHAS mRNA suppression didn’t alter the AHAS locus genetically or epigenetically [41]. After searching published reports, no miRNA has been identified to availably regulate AHAS genes in plants at present.

In this study, an AHAS herbicides tolerant genotype and a sensitive genotype were used for miRNA comparative analysis. A total of 20 small RNA libraries were obtained of these two genotypes at three time points (0h, 24h and 48h) after spraying SU and IMI herbicides, with two replicates. We identified 940 conserved miRNAs and 1515 novel candidate miRNAs in rapeseed using high-throughput sequencing methods combined with computing analysis. A total of 3284 genes were predicted to be targets of these miRNAs, and their functions were shown using GO, KOG and KEGG annotations. The differentiation expression results of miRNAs showed almost twice as many differentiated miRNAs were found in tolerant genotype M342 (309 miRNAs) after SU herbicide application than in sensitive genotype N131 (164 miRNAs). In addition, there were 177 and 296 miRNAs defined as differentiated in sensitive genotype N131 and tolerant genotype M342 in response to SU herbicides. The miR398 family was observed to be associated with AHAS herbicide tolerance because their expression increased in the tolerant genotype but decreased in the sensitive genotype. This study not only provides novel insights into the miRNA content of AHAS herbicides tolerant rapeseed in response to AHAS herbicides but also demonstrates that miRNAs may be involved in AHAS herbicides tolerance.

Small RNA sequencing provides a high-throughput method to identify miRNAs which were easily to analyze due to typical precursor hairpin characteristics. Although other small RNAs like *cis*-trans RNAs and circular RNAs also included in sequencing data, a lack of typical structure and short sequence length decreases the possibility of detecting them effectively using bioinformatics methods. In addition, these sequencing data would be a valuable resource for future small RNA identification and expression detection by computational methods.

## Supporting information

S1 File Table A in S1 File. Selected miRNAs and target genes for qPCR validation. Table B in S1 File. Length distribution of small RNAs. Table C in S1 File. The region assigned for reads and types of small RNAs. Table D in S1 File. Repeat type in reads. Table E in S1 File. The expression of known miRNAs in rapeseed. Table F in S1 File. miRNA families and their expression. Table G in S1 File. Predicted target gene of miRNAs in rapeseed. Table H in S1 File. The information of predicted precursor miRNAs in rapeseed. Table I in S1 File. The information of predicted mature miRNAs in rapeseed. Table J in S1 File. The differentiated miRNAs after spraying SU herbicide in genotype N131. Table K in S1 File. The differentiated miRNAs after spraying SU herbicide in genotype M342. Table L in S1 File. The differentiated miRNAs after spraying IMI herbicide in genotype N131. Table M in S1 File. The differentiated miRNAs after spraying IMI herbicide in genotype M342.
(XLSX)

S2 File The predicted secondary structure for identified novel miRNAs in rapeseed. (ZIP)
