# Supplementary figures and images for: Comparative analysis of miRNAs of two rapeseed genotypes in response to acetohydroxyacid synthase-inhibiting herbicides by high-throughput sequencing
Source: PLoS One. 2017 Sep 26;12(9):e0184917. doi: 10.1371/journal.pone.0184917 (PMC5614533; doi:10.1371/journal.pone.0184917)

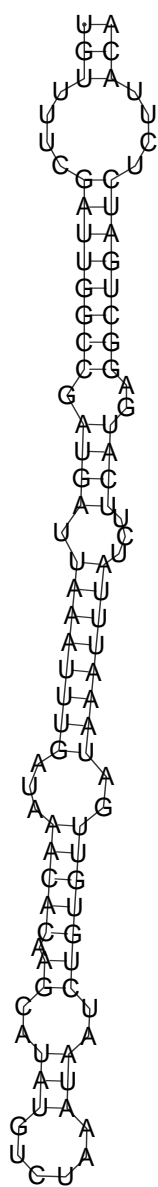

Supplement: S2 File — (ZIP) [file pone.0184917.s002.zip › SecondaryStructure/Bna-MIR-n001.pdf]

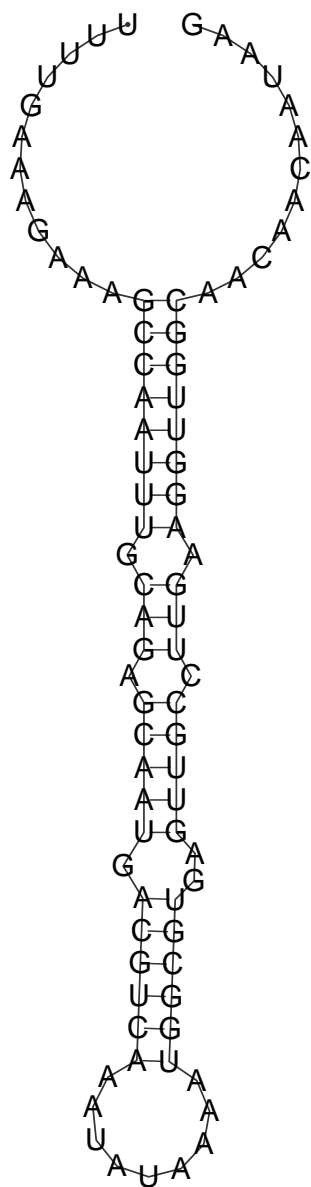

Supplement: S2 File — (ZIP) [file pone.0184917.s002.zip › SecondaryStructure/Bna-MIR-n002.pdf]

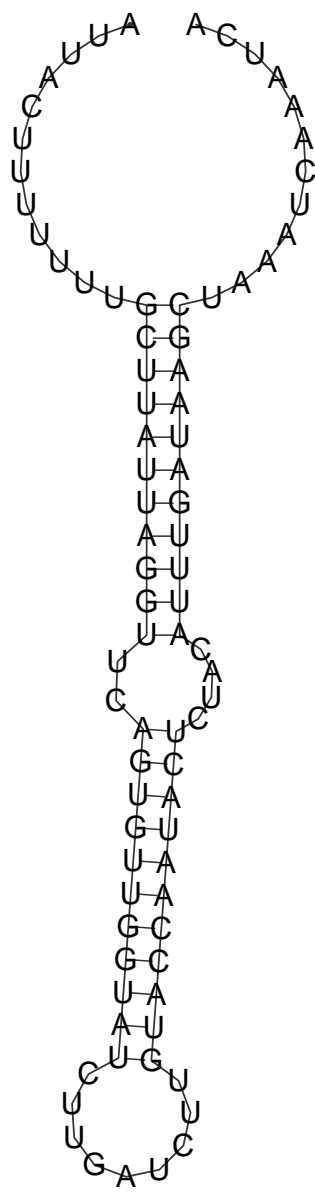

Supplement: S2 File — (ZIP) [file pone.0184917.s002.zip › SecondaryStructure/Bna-MIR-n003.pdf]

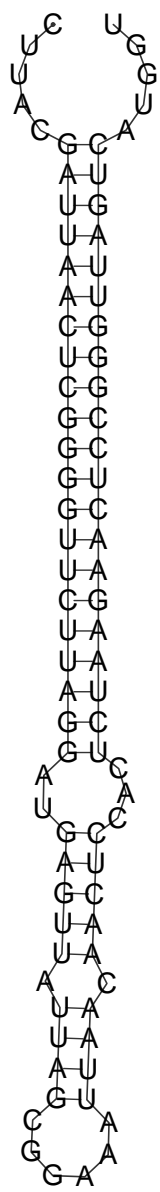

Supplement: S2 File — (ZIP) [file pone.0184917.s002.zip › SecondaryStructure/Bna-MIR-n004.pdf]

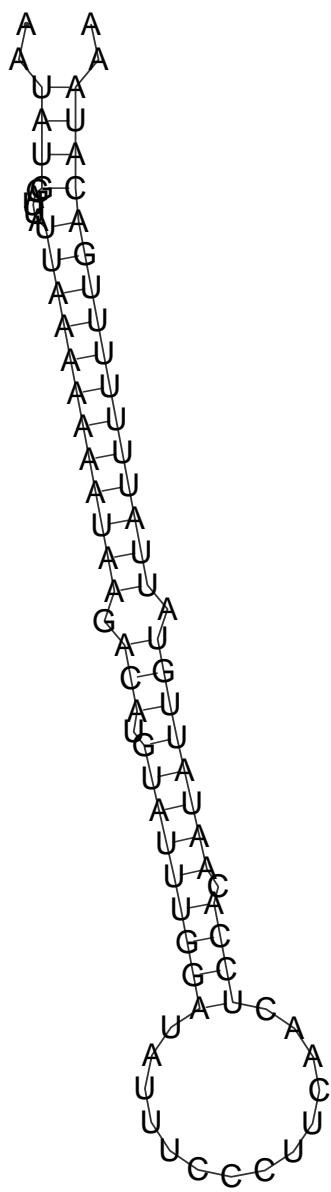

Supplement: S2 File — (ZIP) [file pone.0184917.s002.zip › SecondaryStructure/Bna-MIR-n005.pdf]

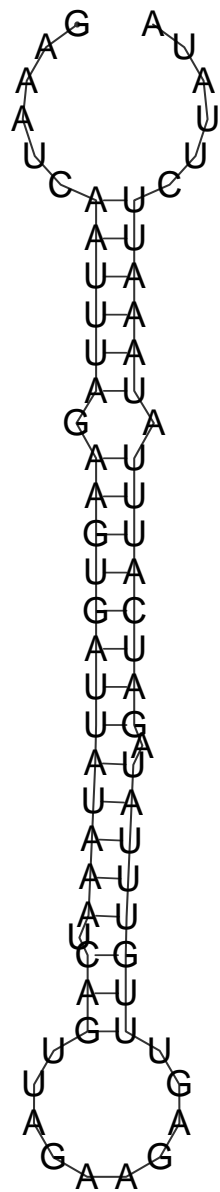

Supplement: S2 File — (ZIP) [file pone.0184917.s002.zip › SecondaryStructure/Bna-MIR-n006.pdf]

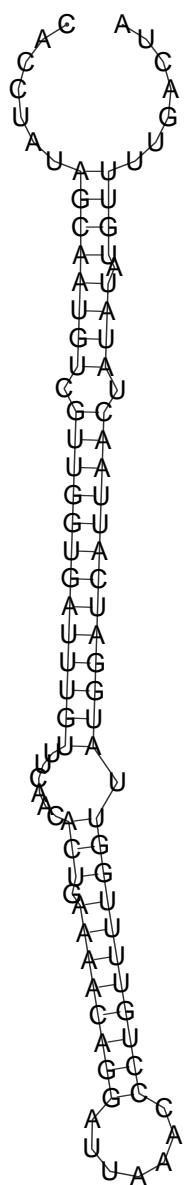

Supplement: S2 File — (ZIP) [file pone.0184917.s002.zip › SecondaryStructure/Bna-MIR-n007.pdf]

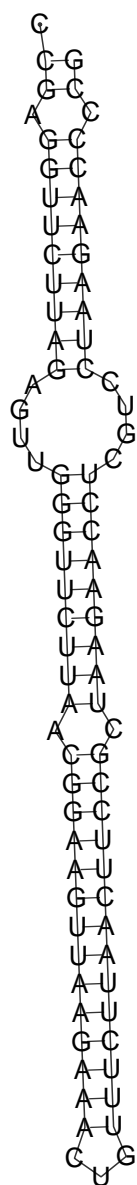

Supplement: S2 File — (ZIP) [file pone.0184917.s002.zip › SecondaryStructure/Bna-MIR-n008.pdf]

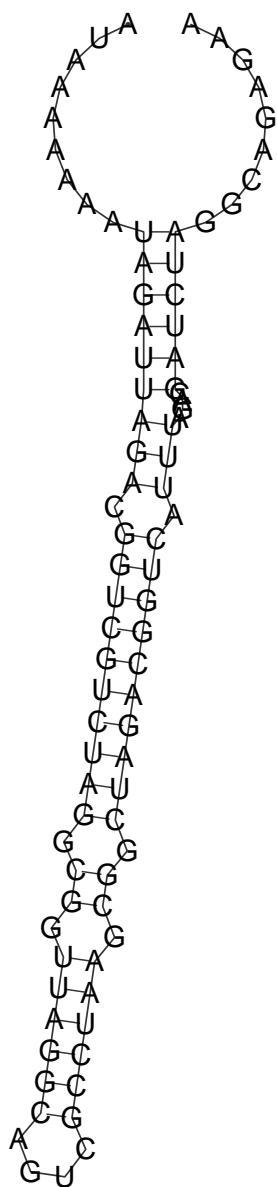

Supplement: S2 File — (ZIP) [file pone.0184917.s002.zip › SecondaryStructure/Bna-MIR-n009.pdf]

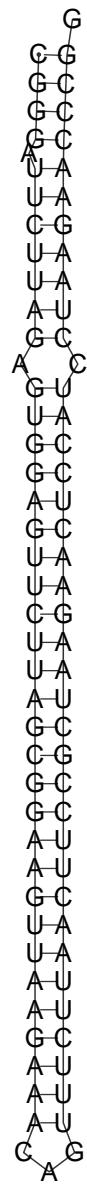

Supplement: S2 File — (ZIP) [file pone.0184917.s002.zip › SecondaryStructure/Bna-MIR-n010.pdf]

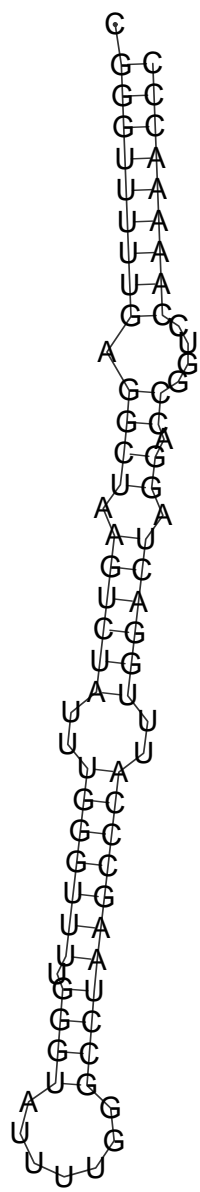

Supplement: S2 File — (ZIP) [file pone.0184917.s002.zip › SecondaryStructure/Bna-MIR-n011.pdf]

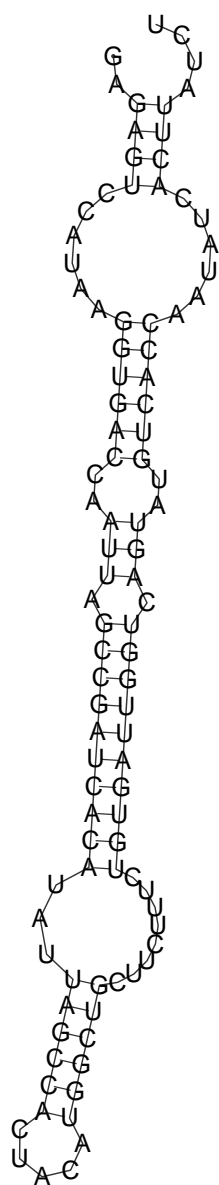

Supplement: S2 File — (ZIP) [file pone.0184917.s002.zip › SecondaryStructure/Bna-MIR-n012.pdf]

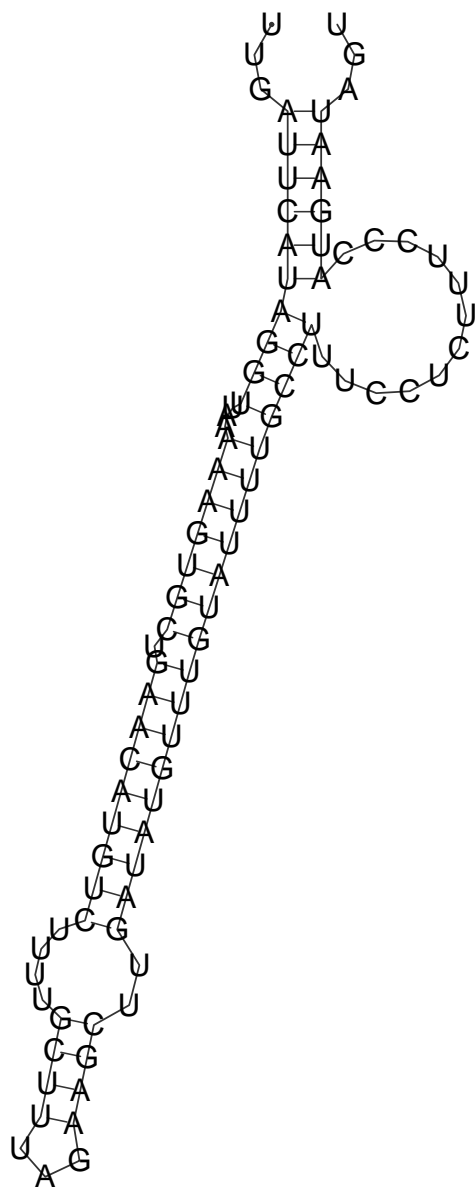

Supplement: S2 File — (ZIP) [file pone.0184917.s002.zip › SecondaryStructure/Bna-MIR-n013.pdf]

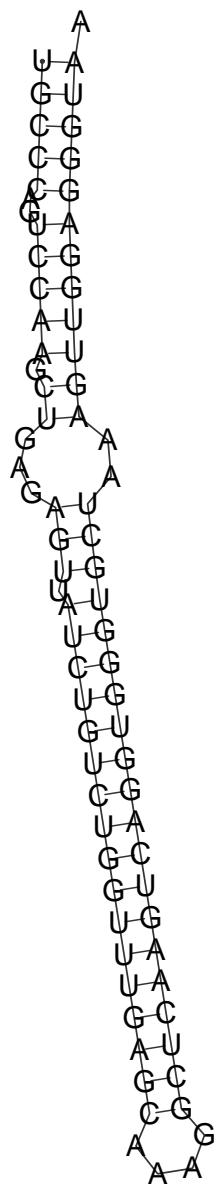

Supplement: S2 File — (ZIP) [file pone.0184917.s002.zip › SecondaryStructure/Bna-MIR-n014.pdf]

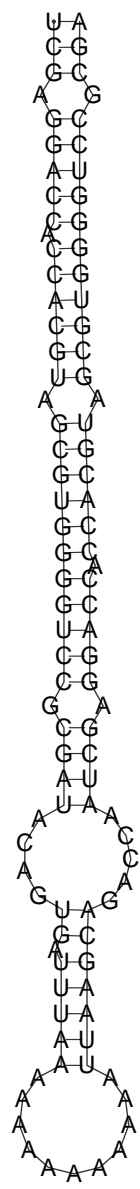

Supplement: S2 File — (ZIP) [file pone.0184917.s002.zip › SecondaryStructure/Bna-MIR-n015.pdf]

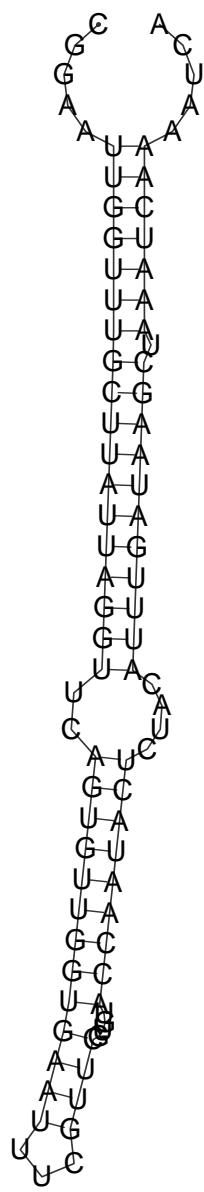

Supplement: S2 File — (ZIP) [file pone.0184917.s002.zip › SecondaryStructure/Bna-MIR-n016.pdf]

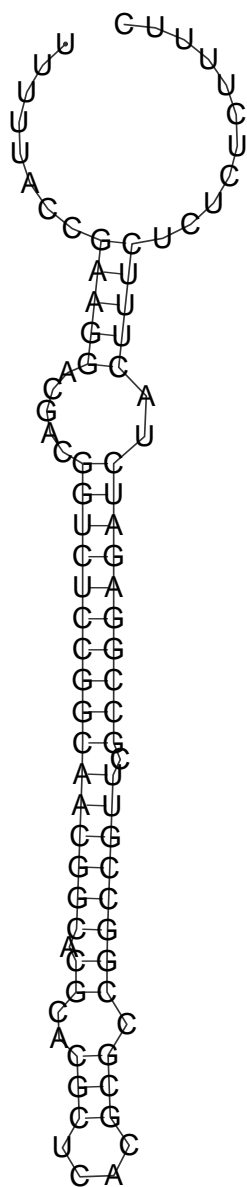

Supplement: S2 File — (ZIP) [file pone.0184917.s002.zip › SecondaryStructure/Bna-MIR-n017.pdf]

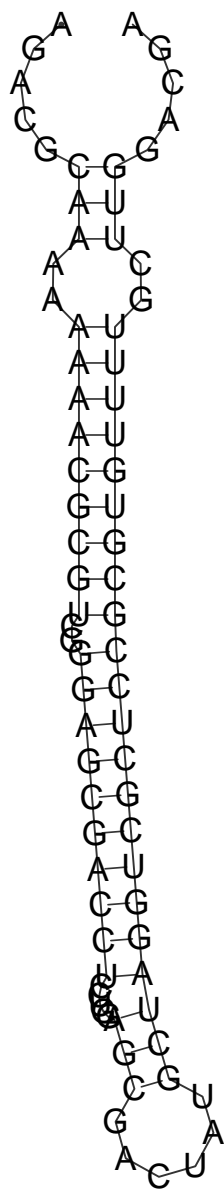

Supplement: S2 File — (ZIP) [file pone.0184917.s002.zip › SecondaryStructure/Bna-MIR-n018.pdf]

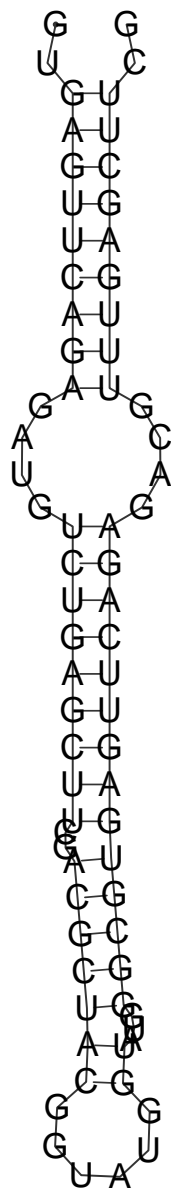

Supplement: S2 File — (ZIP) [file pone.0184917.s002.zip › SecondaryStructure/Bna-MIR-n019.pdf]

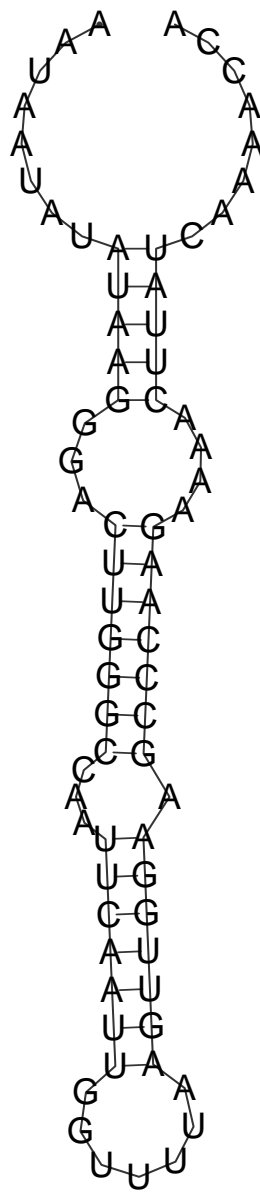

Supplement: S2 File — (ZIP) [file pone.0184917.s002.zip › SecondaryStructure/Bna-MIR-n020.pdf]

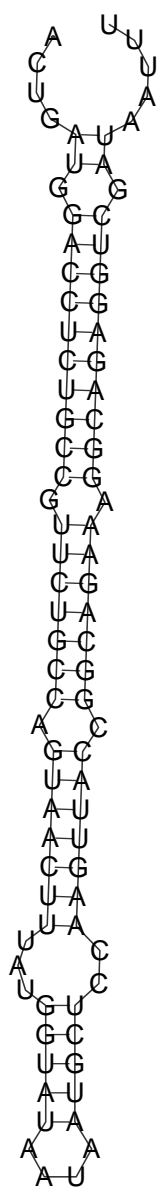

Supplement: S2 File — (ZIP) [file pone.0184917.s002.zip › SecondaryStructure/Bna-MIR-n021.pdf]

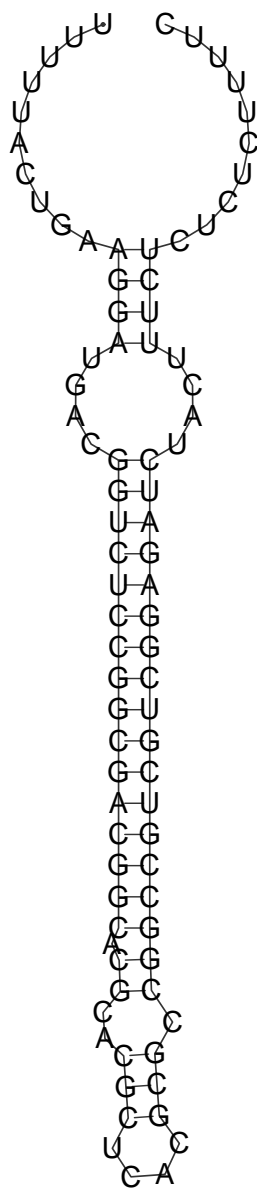

Supplement: S2 File — (ZIP) [file pone.0184917.s002.zip › SecondaryStructure/Bna-MIR-n022.pdf]

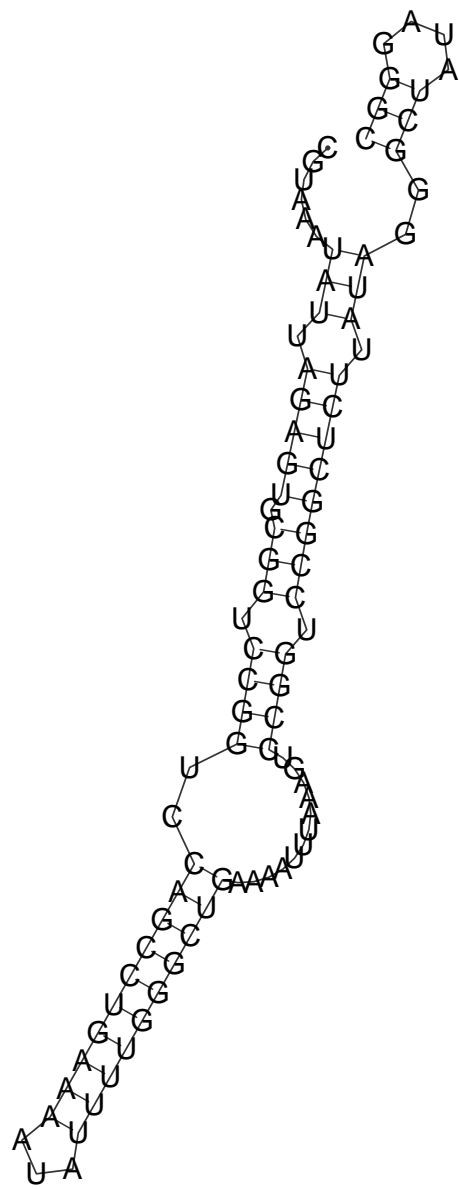

Supplement: S2 File — (ZIP) [file pone.0184917.s002.zip › SecondaryStructure/Bna-MIR-n023.pdf]

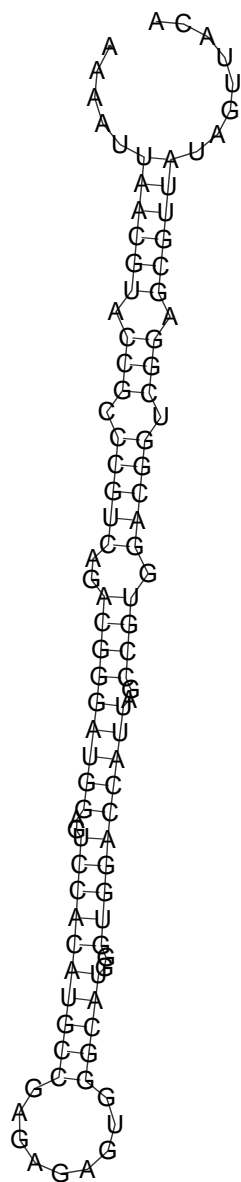

Supplement: S2 File — (ZIP) [file pone.0184917.s002.zip › SecondaryStructure/Bna-MIR-n024.pdf]

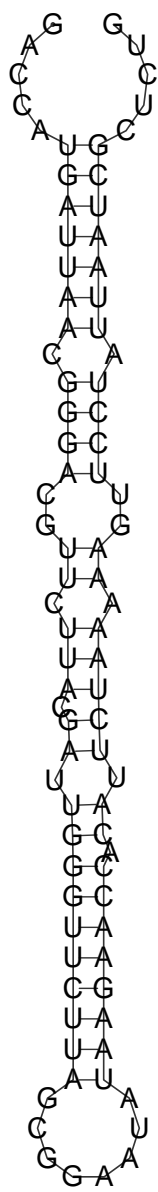

Supplement: S2 File — (ZIP) [file pone.0184917.s002.zip › SecondaryStructure/Bna-MIR-n025.pdf]

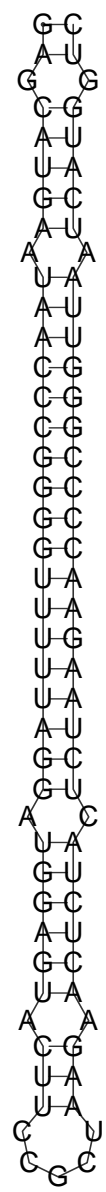

Supplement: S2 File — (ZIP) [file pone.0184917.s002.zip › SecondaryStructure/Bna-MIR-n026.pdf]

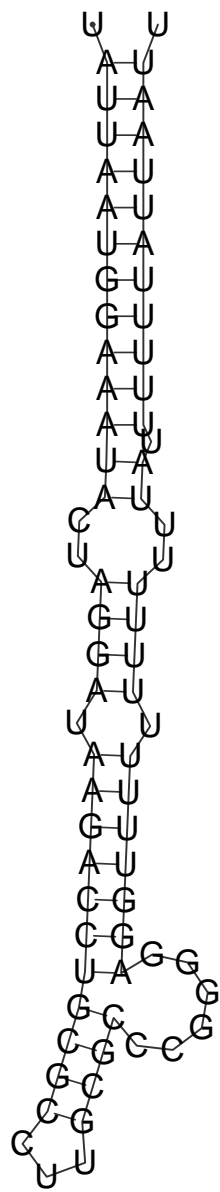

Supplement: S2 File — (ZIP) [file pone.0184917.s002.zip › SecondaryStructure/Bna-MIR-n027.pdf]

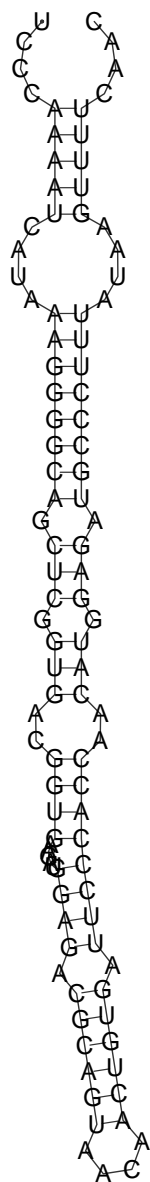

Supplement: S2 File — (ZIP) [file pone.0184917.s002.zip › SecondaryStructure/Bna-MIR-n028.pdf]

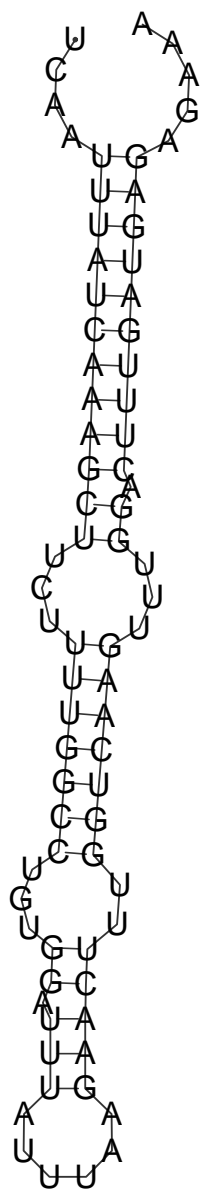

Supplement: S2 File — (ZIP) [file pone.0184917.s002.zip › SecondaryStructure/Bna-MIR-n029.pdf]

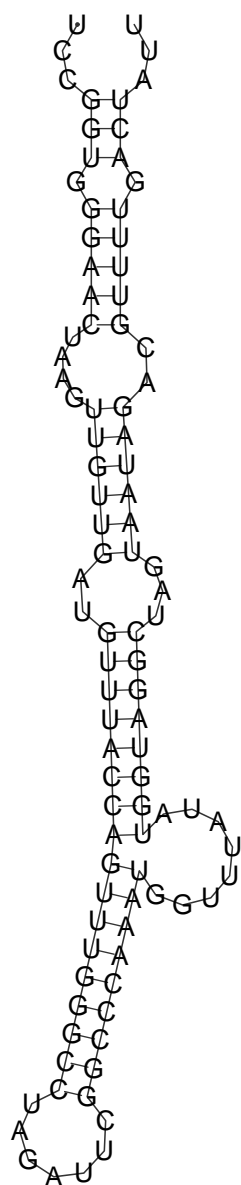

Supplement: S2 File — (ZIP) [file pone.0184917.s002.zip › SecondaryStructure/Bna-MIR-n030.pdf]

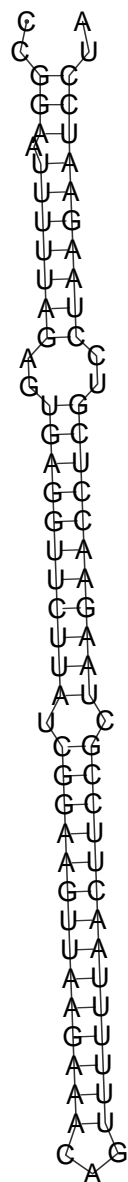

Supplement: S2 File — (ZIP) [file pone.0184917.s002.zip › SecondaryStructure/Bna-MIR-n031.pdf]

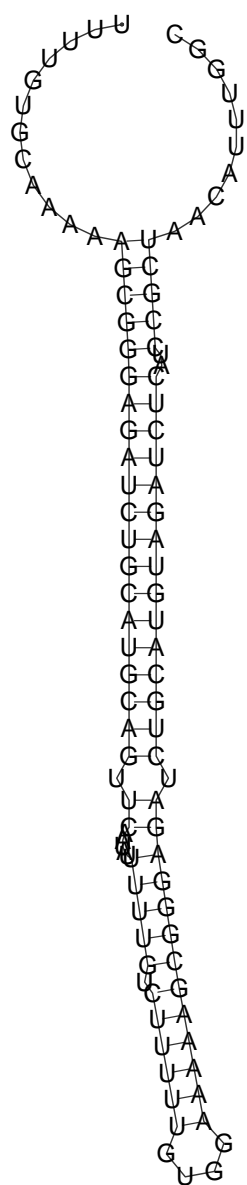

Supplement: S2 File — (ZIP) [file pone.0184917.s002.zip › SecondaryStructure/Bna-MIR-n032.pdf]

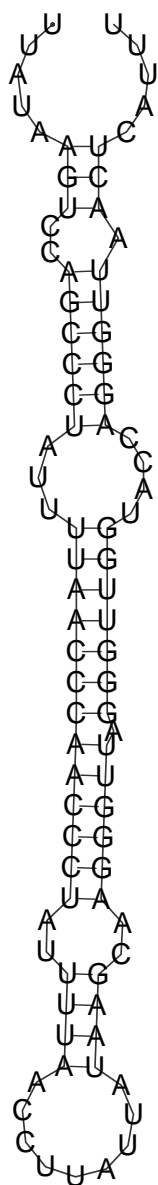

Supplement: S2 File — (ZIP) [file pone.0184917.s002.zip › SecondaryStructure/Bna-MIR-n033.pdf]

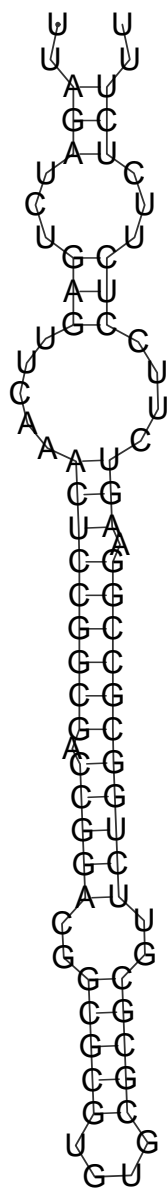

Supplement: S2 File — (ZIP) [file pone.0184917.s002.zip › SecondaryStructure/Bna-MIR-n034.pdf]

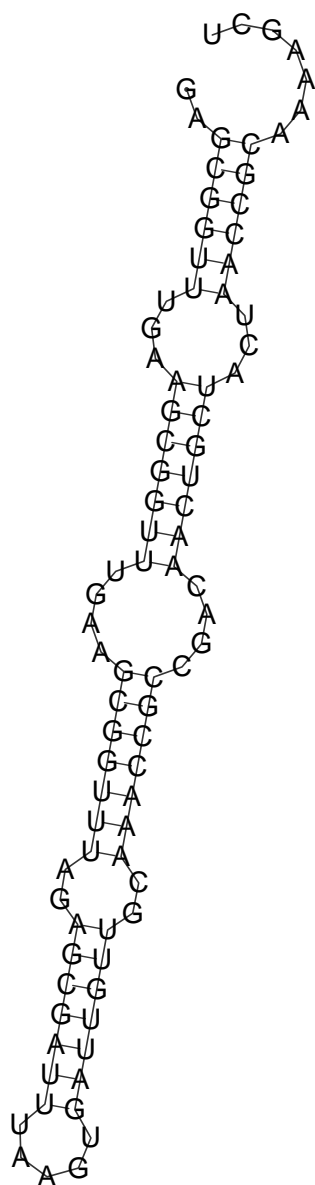

Supplement: S2 File — (ZIP) [file pone.0184917.s002.zip › SecondaryStructure/Bna-MIR-n035.pdf]

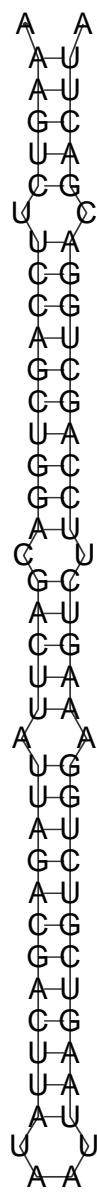

Supplement: S2 File — (ZIP) [file pone.0184917.s002.zip › SecondaryStructure/Bna-MIR-n036.pdf]

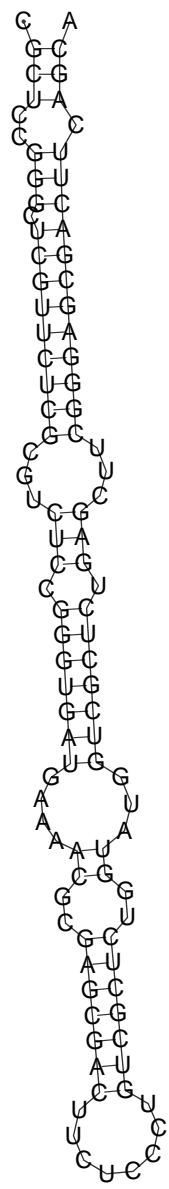

Supplement: S2 File — (ZIP) [file pone.0184917.s002.zip › SecondaryStructure/Bna-MIR-n037.pdf]

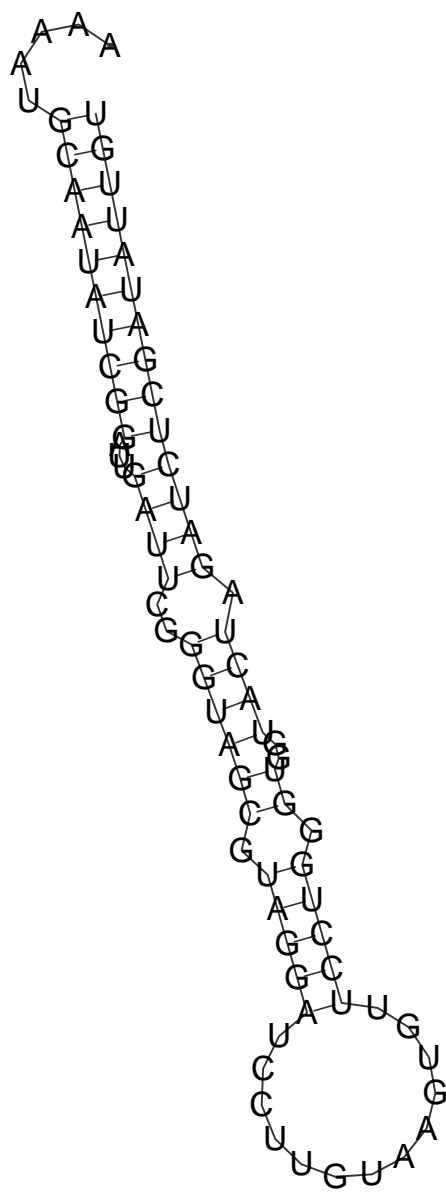

Supplement: S2 File — (ZIP) [file pone.0184917.s002.zip › SecondaryStructure/Bna-MIR-n038.pdf]

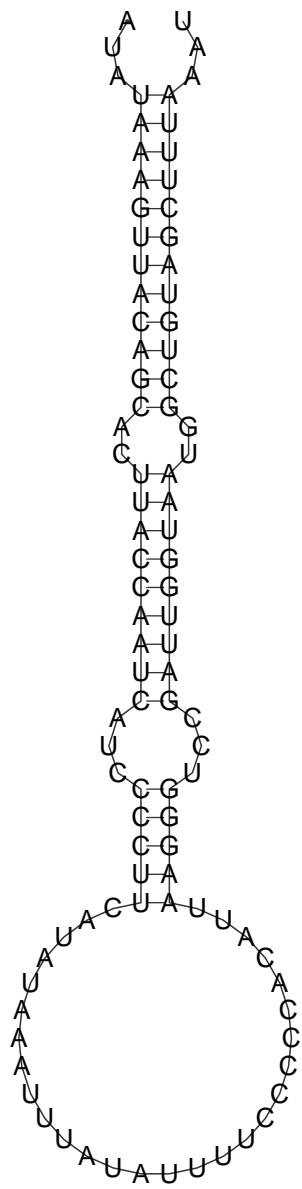

Supplement: S2 File — (ZIP) [file pone.0184917.s002.zip › SecondaryStructure/Bna-MIR-n039.pdf]
